# Efficient induction of pancreatic alpha cells from human induced pluripotent stem cells by controlling the timing for BMP antagonism and activation of retinoic acid signaling

**DOI:** 10.1371/journal.pone.0245204

**Published:** 2021-01-11

**Authors:** Shigeharu G. Yabe, Satsuki Fukuda, Junko Nishida, Fujie Takeda, Kiyoko Nashiro, Hitoshi Okochi

**Affiliations:** Department of Regenerative Medicine, Research Institute, National Center for Global Health and Medicine, Tokyo, Japan; Purdue University, UNITED STATES

## Abstract

Diabetes mellitus is caused by breakdown of blood glucose homeostasis, which is maintained by an exquisite balance between insulin and glucagon produced respectively by pancreatic beta cells and alpha cells. However, little is known about the mechanism of inducing glucagon secretion from human alpha cells. Many methods for generating pancreatic beta cells from human pluripotent stem cells (hPSCs) have been reported, but only two papers have reported generation of pancreatic alpha cells from hPSCs. Because NKX6.1 has been suggested as a very important gene for determining cell fate between pancreatic beta and alpha cells, we searched for the factors affecting expression of NKX6.1 in our beta cell differentiation protocols. We found that BMP antagonism and activation of retinoic acid signaling at stage 2 (from definitive endoderm to primitive gut tube) effectively suppressed NKX6.1 expression at later stages. Using two different hPSCs lines, treatment with BMP signaling inhibitor (LDN193189) and retinoic acid agonist (EC23) at Stage 2 reduced NKX6.1 expression and allowed differentiation of almost all cells into pancreatic alpha cells *in vivo* after transplantation under a kidney capsule. Our study demonstrated that the cell fate of pancreatic cells can be controlled by adjusting the expression level of NKX6.1 with proper timing of BMP antagonism and activation of retinoic acid signaling during the pancreatic differentiation process. Our method is useful for efficient induction of pancreatic alpha cells from hPSCs.

## Introduction

The number of diabetes mellitus patients is increasing in the world. Hyperglycemia is a feature of diabetes mellitus caused by insufficient insulin secretion or insulin resistance. However, diabetic patients often show paradoxical hyperglucagonemia; this mechanism has not yet been elucidated [1–3]. Insulin secreted by pancreatic beta cells lowers blood glucose level; in contrast, glucagon secreted by the pancreatic alpha cells raises the level of blood glucose, thus maintaining the blood glucose level within the normal range by an exquisite balance between these two hormones [4]. Hypoglycemia is also a problem in diabetic patients using insulin replacement therapy; in particular severe hypoglycemia can lead to death in type 1 diabetes mellitus [5]. Dysfunction of pancreatic alpha cells in type 1 diabetes mellitus is reported to be responsible for such hypoglycemia [6–8]. Moreover, glucagon messaging from pancreatic alpha cells to pancreatic beta cells is important for the determination of glycemic set points [9]. Although this hormone network between pancreatic beta cells and alpha cells is critical for understanding the pathophysiology of the diabetes mellitus, little is known about human alpha cells because of the lack of methods for isolating them.

Recently, cell therapies using human pluripotent stem cells (hPSCs) have been developed, and a great deal of effort has been put into generation of pancreatic beta cells [10–20]. Hyperglycemia in diabetic model mice has been ameliorated by transplanting hPSC-derived pancreatic beta cells into them [16,17,20]. Although numerous papers have described methods for hPSCs-beta cell differentiation, only two papers describing differentiation protocols for pancreatic alpha cells from hPSCs are reported; one is from Kieffer’s group [21] and the other is very recently from Melton`s group [22]. NKX6.1 expression was shown to be important in determining cell fate between pancreatic beta and alpha cells; cells expressing high levels of NKX6.1 differentiated into pancreatic beta cells, but cells expressing low levels differentiated into pancreatic alpha cells [15]. Based on this information, we focused on the expression of NKX6.1 during the pancreatic islet differentiation process from human iPS cells and found key molecules for the efficient induction of pancreatic alpha cells. We demonstrated that treatment with LDN193189 (BMP antagonist) and EC23 (retinoic acid analog) during the primitive gut tube stage reduced NKX6.1 expression at later stages and eventually promoted induction of pancreatic alpha cells.

## Methods

### Culture of undifferentiated hiPSCs

The TkDN4-M (4M) hiPSCs line was a kind gift from Dr. M Ohtsu at The Institute of Medical Science, The University of Tokyo. 4M cells were cultured as previously described with minor modifications [23]. They were maintained on mitomycin C (MMC; FUJIFILM Wako Pure Chemical Corporation, Osaka, Japan)-treated SNL feeder cells in hiPSCs medium (DMEM/Ham`s F12 (FUJIFILM Wako) supplemented with 20% Knockout Serum Replacement (KSR; GIBCO BRL, Palo Alto, CA, USA), 1x MEM nonessential amino acids (FUJIFILM Wako), 0.5x penicillin streptomycin(PS; FUJIFILM Wako), 55μM 2-melcaptoethanol (2ME Gibco) and 7.5μg/ml basic fibroblast growth factor (FGF2; Peprotech, Rocky Hill, NJ, USA). For passage, 4M colonies were detached using CTK solution, chipped by pipetting, and seeded onto MMC-treated SNL feeder (ECACC, Salisbury, UK) in hiPSCs medium once a week. The 15M63 hiPSCs line was kindly provided from the Center for iPS Cell Research and Application of Kyoto University (Kyoto, Japan) and maintained on vitronectin (Invitrogen, CA, USA) coated dishes in StemFit. For passage, 15M63 was dissociated with accutase (Innovative Cell Technologies, San Diego, USA) by pipetting and seeded on vitronectin coated dishes in StemFit (Ajinomoto, Tokyo, Japan) supplemented with 10μM Y27632 (Cayman Chemical, Ann Arbor, MI, USA).

### Differentiation into pancreatic beta or alpha cells

Differentiation of hiPSCs was induced in suspension culture using bioreactors. Undifferentiated 4M cells were detached from the seeder cells with CTK solution and dissociated into single cells with Accumax (Innovative Cell Technologies). Then cells were seeded at a density of 1x10^6^ cells/ml in a spinner type bioreactor (ABLE, Tokyo, Japan) containing 30ml of mTeSR1 (VERITAS, Tokyo, Japan) with 10μM ROCK inhibitor. The next day, culture medium was exchanged for fresh mTeSR1. One day later, culture medium was exchanged for hiPS medium, and differentiation was started the next day. Undifferentiated 15M63 cells were dissociated with Accutase and seeded at a density of 1x10^6^ cells/ml in the spinner type bioreactor containing 30ml of StemFit with 10μM ROCK inhibitor. The next day, culture medium was exchanged for fresh mTeSR1. One day later, culture medium was exchanged with hiPS medium and differentiation was started at next day.

At stage 1, Spheroids were cultured for 2 days in RPMI 1640 (FUJIFILM Wako) supplemented with 0.25% bovine serum albumin (BSA; Sigma, USA), 0.4x PS (FUJIFILM Wako), 1mM sodium pyruvate (FUJIFILM Wako), 1xNEAA, 80ng/mL recombinant human activin A (Peprotech), 55μM 2-ME, 50ng/ml FGF2, 20ng/ml recombinant bone morphogenetic protein 4 (BMP4; Peprotech) and 3μM CHIR99021 (Biovision, Milpitas, CA, USA). Then the spheroids were cultured for 1 day in RPMI 1640 supplemented with 1:200 Insulin, Transferrin, Selenium, Ethanolamine Solution (ITS-X; Gibco), 0.25% bovine serum albumin (BSA; Sigma), 0.4x penicillin and streptomycin, 1mM sodium pyruvate, 1x NEAA, 80ng/mL recombinant human activin A (Peprotech), and 55μM 2-ME. Next, the spheroids were cultured for 1 day in RPMI 1640 (FUJIFILM Wako) supplemented with 0.25% bovine serum albumin (BSA; Sigma), 0.4x PS, 1mM sodium pyruvate, 1x NEAA, 80ng/mL recombinant human activin A (Peprotech) and 55μM 2-ME.

At stage 2, spheroids were cultured for 3 days in RPMI 1640 supplemented with 0.25% BSA, 1mM sodium pyruvate, 1x NEAA, 0.4x PS, and 50ng/ml recombinant human FGF7 (Peprotech), 1:200 ITS-X. For LDN193189 and EC23 treatment, 0.04μM LDN193189, 1μM EC23, 2.5μM Y27632 and 5μg/ml heparin were added to the medium. The medium was changed on the third day We named these LDN193189 and EC23 treated cells as LEN + EC cells.

At stage 3, spheroids were cultured in DMEM (8mM glucose) supplemented with 0.25% BSA, 0.4x PS, 1x NEAA, 50ng/ml FGF7, 1:200 ITS-X, 0.5 μM EC23 (Santa Cruz Biotechnology, USA), 0.2μM LDN 193189 (Cayman Chemical), 0.3μM indolactam V (ILV; Cayman Chemical), and 0.25μM SANT1 (Cayman Chemical) for 4 days. During stage 3, the medium was changed every 2 days.

At stage 4, spheroids were cultured in DMEM (8mM glucose) supplemented with 0.25% BSA, 0.4xPS, 1x NEAA, 50ng/ml recombinant human FGF10 (Peprotech), 1% B27 supplement, 1:400 ITS-X, 0.04μM EC23, 0.2μM LDN 193189, 0.3μM ILV, and 0.25μM SANT1 and 5μM ZnSO_4_ (Sigma) for 3 days. The medium was changed on the third day.

At stage 5, spheroids were cultured in DMEM (20mM glucose) supplemented with 0.25% BSA, 0.4xPS, 1x NEAA, 20ng/ml recombinant human EGF (Peprotech), 1% B27 supplement, 1:400 ITS-X, 0.02μM EC23, 0.2μM LDN 193189, 0.25μM SANT1, 10μM Rep Sox, 5μM ZnSO_4_, 50ng/ml exendin-4 (Abcam), 10μg/ml heparin (Sigma), 10μM Y27632, 0.5μM DBZ (Cayman Chemical) and 5mM Nicotinamide (Sigma) for 7 days. For the LDN + EC cells, the concentration of Rep Sox was 1μM. During stage 5, the medium was changed every 2 days.

At stage 6, spheroids were cultured in DMEM (20mM glucose) supplemented with 0.25% BSA, 0.4xPS, 1x NEAA, 1% B27 supplement, 1:400 ITS-X, 10μM Rep Sox, 5μM ZnSO_4_, 50ng/ml exendin-4, 10μg/ml heparin, 5mM Nicotinamide, 1μM R428 (Cayman Chemical) and 5μM forskolin (FUJIFILM Wako) for 10 days. For the LDN + EC cells, the concentration of Rep Sox was 1μM. During stage 6, the medium was changed every 2 days.

### Encapsulation of differentiated cells into alginate fiber

Differentiated cells were embedded in alginate fibers previously described (Fukuda et al., 2019) [24].

### Quantitative reverse transcription polymerase chain reaction (qRT-PCR)

Extraction and purification of total RNA were carried out using RNAiso. Then cDNA was synthesized with random nonamer and oligo dT(18) using PrimeScript II reverse transcriptase. qPCR was performed using GoTaq qPCR mix and run on a CFX96 Touch Deep Well (Bio-Rad, Hercules, CA, USA). Relative quantification was conducted by standard curve method, and the expression levels of target genes were normalized against that of the reference gene. Ornithine decarboxylase antizyme (OAZ1) was used as the reference gene.

### Immunochemistry

Differentiated cells were fixed with 4% paraformaldehyde at RT for 20 min, rinsed with PBS twice, and next with MilliQ water; then they were stored in 70% ethanol. Then these cells were embedded in paraffin and sectioned. Hematoxylin and eosin staining was performed according to the standard protocol. Immunofluorescent staining was performed with primary antibodies for target genes and fluorescein conjugated secondary antibodies. Primary antibodies included mouse anti-NKX6.1, 1:100 (DSHB, University of Iowa), rat anti-C-peptide, 1:200 (DSHB), rabbit anti-proglucagon 1:300 (CST), and rabbit anti-glucagon 1:300 (Thermo Fisher). The following secondary antibodies were used: Alexa Fluor 488-conjugated donkey anti-goat IgG (Invitrogen), Alexa Fluor 488-conjugated donkey anti-mouse IgG (Invitrogen), Alexa 594-conjugated goat anti-rat IgG (Invitrogen) and Alexa Fluor 488-conjugated goat anti-rabbit IgG (Invitrogen). The positive and surviving rate in each image were calculated with Metamorph image analysis software (Molecular Devices, CA, USA).

### Animal studies

All animal experiments were approved by the Animal Care and Use Committee in the National Center for Global Health and Medicine, and carried out in accordance with institutional procedures, national guidelines, and the relevant national laws on the protection of animals. Eight-week-old male NOD/SCID mice were obtained from Japan Clea, and maintained on a 12‐hour light/dark cycle with ad libitum access to drinking water and standard irradiated diet. These mice were housed for 1 week before transplantation and randomly transplanted with differentiated cells. Mice were anesthetized with a mixture of medetomidine (Nippon Zenyaku Kogyo, Fukushima, Japan), midazolam (Sando, Tokyo, Japan), and butorphanol (Meiji Seika Pharma, Tokyo, Japan) and 6 x 10^6^ differentiated cells were transplanted under the kidney capsule (4M: n = 4, 15M63: n = 4). Mice were anesthetized with inhalable isoflurane (Pfizer, NY, USA) and alginate fiber containing 6 x 10^6^ differentiated cells were transplanted into the peritoneal cavity (4M: n = 4, 15M63: n = 4). Transplanted kidney and alginate fiber were randomly retrieved from mice at 3 or 6 weeks after implantation and used for hormone secretion assays and histochemical analysis. All mice were anesthetized with inhalable sevoflurane (Maruishi Pharmaceutical, Osaka, Japan) and euthanized by cervical dislocation, at the time of sacrifice. No mice reached the experimental endpoint. Although investigators could not be blinded in these experiments, obtained data were evaluated by both SGY and HO to avoid arbitrary interpretation.

### Static hormone secretion experiment

Retrieved kidney was cut to remove most of the parenchymal tissue; the remaining tissue was washed with wash medium (DMEM containing 10 mM HEPES and 0.1% BSA) and preincubated at 37°C for 30 min. Then it was incubated with 1 ml of DMEM (1mM glucose) containing 10 mM HEPES and 0.1% BSA at 37°C for 30 min. After collection of supernatant, the sample was rinsed with wash medium and incubated in 1 ml of DMEM (1 mM glucose) containing 10 mM HEPES, 0.1% BSA and 30 mM arginine at 37°C for 30 min. Medium was harvested, and the sample was washed again and incubated in 1 ml of DMEM (6 mM glucose) containing 10 mM HEPES and 0.1% BSA at 37°C for 30 min. Supernatant was recovered, and the sample was washed once more and incubated in 1 ml of DMEM (6 mM glucose) containing 10 mM HEPES, 0.1% BSA and 30 mM arginine at 37°C for 30 min.

### Statistical analysis

Data are expressed as the mean±SD. For comparisons of discrete data sets, unpaired Student’s t-tests were used. Two-tailed P< 0.05 was considered significant. All calculation were performed with Microsoft Excel.

### Measurement of human insulin C-peptide and glucagon

Human insulin C-peptide and glucagon in media were measured using a human C-peptide ELISA kit (Mercodia) and a glucagon ELISA kit (Mercodia) according to the manufacturer`s protocol.

## Results

### BMP signaling antagonism effectively downregulates NKX6.1 expression

**Fig 1:**
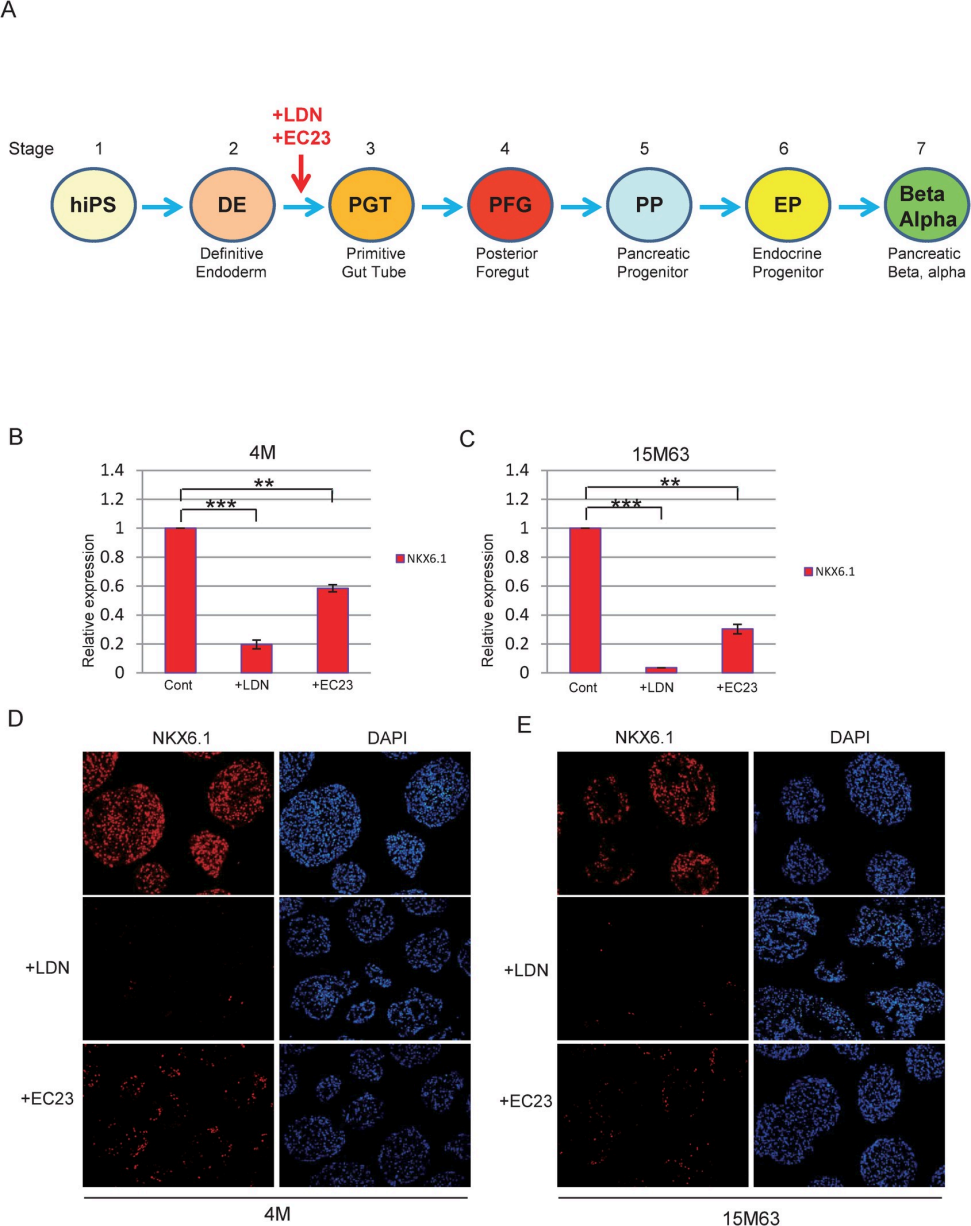
Scheme of the differentiation process and effects of LDN193189 and EC23. (A) Scheme of the stepby-step differentiation protocol. (B, C) Analysis of NKX6.1 mRNA expression in cells treated with LDN193189 or EC23 by qPCR B; 4M, C; 15M63. N = 2 each. **P<0.01, ***P<0.001.(D, E) Analysis of NKX6.1 protein expression in cells treated with LDN193189 or EC23 by immunocytochemistry. D; 4M, E; 15M63.

Rezania et al. reported that hES cell-derived NKX6.1-expressing progenitor cells matured into insulin-secreting cells in vivo, while NKX6.1 negative or low-expressing cells differentiated into pancreatic alpha cells (Rezania et al., 2013). Because NKX6.1 expression is suggested to be very important for determination of the identity of pancreatic beta and alpha cells, we explored which factors affected NKX6.1 expression in our differentiation protocols using two iPSCs lines, TkDN4-M (4M) and 15M63. First, we focused on the regulation of BMP and retinoic acid signaling at stage 2 after definitive endoderm [Fig 1A]. Addition of LDN193189 (BMP signaling antagonist) at stage 2 greatly down regulated NKX6.1 mRNA expression at the later stage 7 [Fig 1B and 1C]. Activation of retinoic acid signaling by adding EC23 (retinoic acid analog) also downregulated NKX6.1 mRNA expression, but to a lesser extent than LDN193189 [Fig 1B and 1C]. Immunocytochemical analysis revealed that LDN193189 or EC23 downregulated NKX6.1 protein expression [Fig 1D and 1E]. Next, in order to examine whether or not repression of NKX6.1 expression was caused by a specific effect of LDN193189, we treated cells with another BMP antagonist, dorsomorphin, which also reduced the NKX6.1 expression to almost the same level as LDN193189 [S1A and S1B Fig], suggesting that the NKX6.1 suppression was caused by a general BMP antagonism. We further investigated whether addition of BMP4 conversely enhanced NKX6.1 expression, but did not up-regulate NKX6.1 expression [S1C and S1D Fig]. These results indicate that inhibition of BMP signaling and activation of retinoic acid signaling at stage 2 lead to reduction of NKX6.1 expression at later stages.

### Addition of both LDN193189 and EC23 effectively suppressed NKX6.1 expression

**Fig 2:**
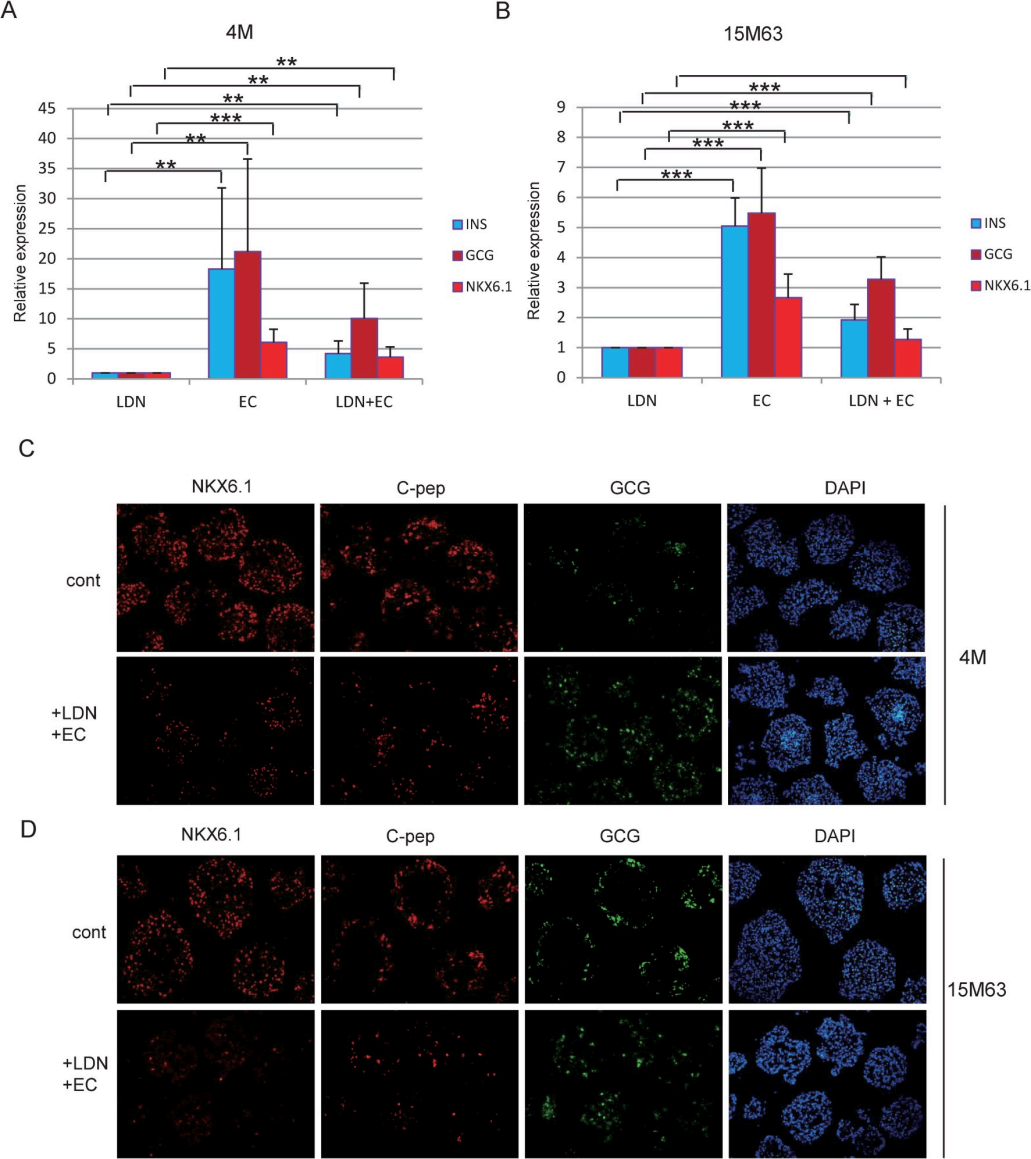
Effects of LDN193189 or EC23 or both LDN193189 + EC23 on expressions of NKX6.1, insulin and glucagon. (A, B) Analysis of mRNA expression in cells treated with LDN or EC or both LDN193189 + EC23 by qPCR. A; 4M, B; 15M63, N = 5 each. **P<0.01, ***P<0.001. ns: not significant. (C, D) Analysis of protein expression in cells treated with LDN19319 + EC23 by immunocytochemistry. C; 4M, D; 15M63. In the case of 4M, positive staining rates were 75.9 ± 8.3% and 4.8 ± 3.3% for NKX6.1 (P<0.001), 38.8 ± 2.6% and 14.8 ± 2.2% for C-peptide (P<0.001), 4.8 ± 3.3% and 33.4 ± 7.7% for GCG (P<0.001), in control and LDN + EC cells respectively. In the case of 15M63, positive staining rates were 46.0 ± 5.0% and 2.8 ± 0.7% for NKX6.1 (P<0.001), 25.9 ± 1.5% and 3.5 ± 0.6% for C-peptide (P<0.001), 14.8 ± 1.3% and 14.8 ± 1.3% for GCG (P>0.5), in control and LDN + EC cells respectively. N = 3 each.

Next, we wanted to know whether LDN193189 or EC23 promoted differentiation of pancreatic alpha cells from hiPSCs. We first compared the effects of treatment with LDN193189, EC23, or a combination of LDN193189 and EC23 by measuring the gene expressions of INS, GCG and NKX6.1. Although insulin, glucagon and NKX6.1 expressions were increased in LDN193189 + EC23-treated cells compared with LDN193189-treated cells, the relative expression level of glucagon was higher than that of insulin or NKX6.1 in LDN193189 + EC23-treated cells. [Fig 2A and 2B]. Moreover, LDN193189-treated spheroids were fragile and easy to break open compared with LDN193189 + EC23 treated spheroids especially in 4M [S2 Fig] From these results, we adopted a protocol that added LDN19389 + EC23 at stage 2 to differentiate pancreatic alpha cells and named these LDN19389 + EC23 treated cells as LDN + EC cells.

We next used immunocytochemistry to examine the protein expressions of NKX6.1, insulin and glucagon in LDN + EC cells. We confirmed that NKX6.1 positive cells were remarkably reduced in LDN + EC cells (4.8 ± 3.3% in 4M, 2.8 ± 0.7% in 15M63) compared with control cells in both 4M and 15M63 (75.9 ± 8.3% in 4M, 46.0 ± 5.0% in 15M63) [Fig 2C and 2D]. We observed that insulin positive cells were moderately reduced in LDN + EC cells in 4M (14.8 ± 2.2% in LDN + EC, 38.8 ± 2.6% in control) [Fig 2C] and markedly decreased in 15M63 (3.5 ± 0.6% in LDN + EC, 25.9 ± 1.5% in control) [Fig 2D]. Although the rates of GCG positive cells did not differ between LDN + EC cells and control cells for 15M63 (14.0 ± 1.0% in LDN + EC, 14.8 ± 1.3% in control) [Fig 2D], an apparent increase of GCG positive cells was observed in LDN +EC cells for 4M (33.4 ± 7.7% in LDE + EC, 4.8 ± 3.3% in control) [Fig 2C].

### Temporal gene expression analysis of LDN + EC cells

**Fig 3:**
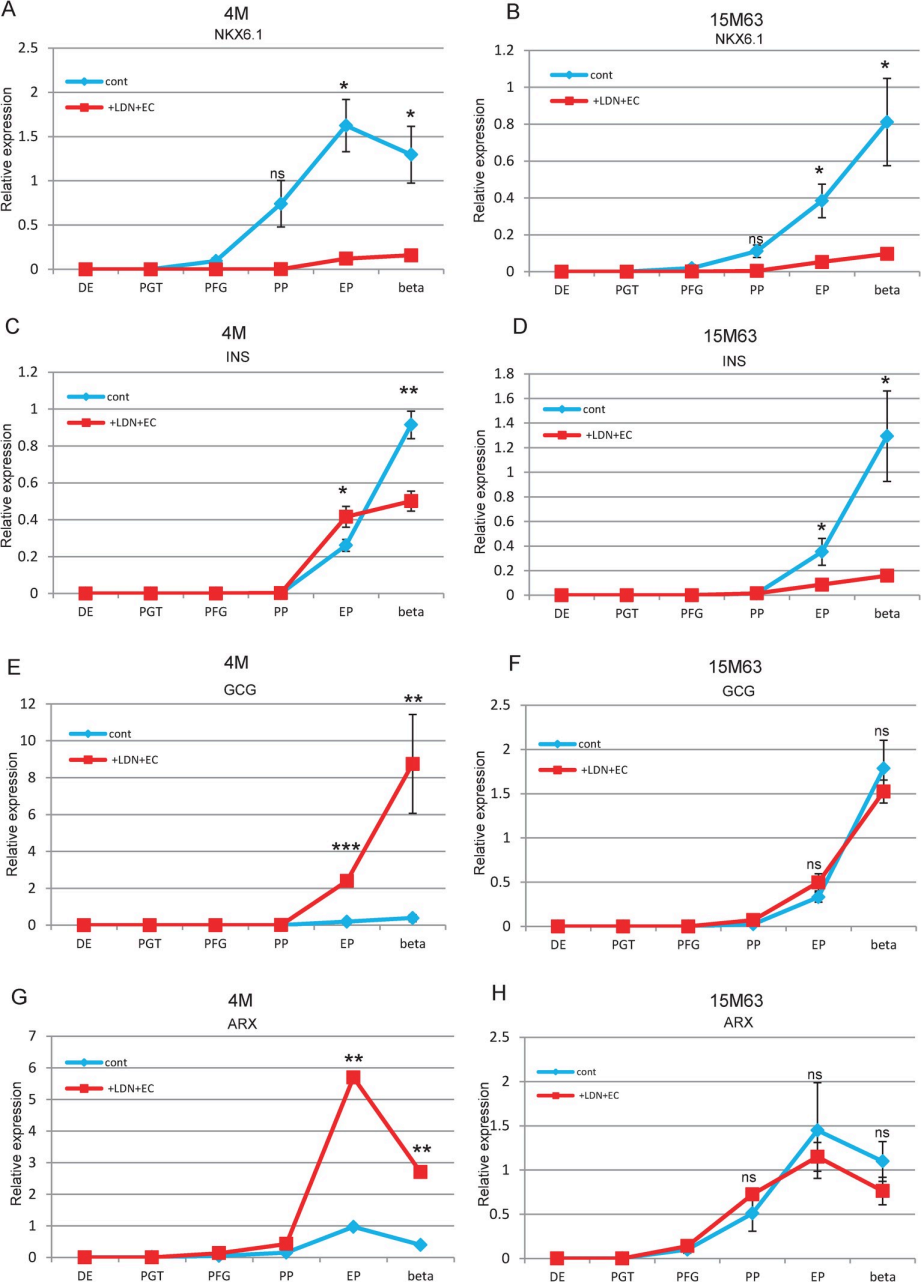
Temporal gene expression patterns analyzed by qPCR (A, B) Temporal expression pattern of NKX6.1. A; 4M, B; 15M63 (C, D) Temporal expression pattern of insulin. C; 4M, D; 15M63 (E, F) Temporal expression pattern of glucagon. E; 4M, F: 15M63 (G, H) Temporal expression pattern of ARX. G; 4M, H: 15M63, Experiments were performed three times. *P<0.05, **P<0.01, ***P<0.001. ns: not significant.

We next compared temporal gene expression during the differentiation process between control and LDN + EC cells. NKX6.1 expression was detected at PFG and gradually increased toward the later stages in control cells. In contrast, NKX6.1 expression barely increased during the entire differentiation process in LDE + EC cells [Fig 3A and 3B]. As for 4M, insulin expression was upregulated at the EP stage in both control and LEN + EC cells, and this expression was further enhanced at the beta stage in control cells but not in LDN + EC cells [Fig 3C]. GCG expression was detected at the EP stage and remarkably increased at the beta stage in LDN + EC cells. In contrast, GCG expression was at an extremely low level during the entire differentiation process in control cells [Fig 3E]. As for 15M63, insulin expression started at the EP stage and was further elevated at the beta stage in control cells, but barely increased in LDN + EC cells [Fig 3D]. GCG expression was noted at the EP stage and up-regulated at the beta stage at similar levels in both control and LDN + EC cells [Fig 3F]. The expression patterns of ARX were similar to these of GCG in both 4M and 15M63 [Fig 3G and 3H]. These results indicate that the expression pattern of NKX6.1 is similar between 4M and 15M63, but the expression patterns of insulin, GCG, and ARX differ between them. These data were consistent with the protein expression patterns obtained by immunocytochemistry, as shown in Fig 2C and 2D.

### Hormone secretion experiments for LDN + EC cells

**Fig 4:**
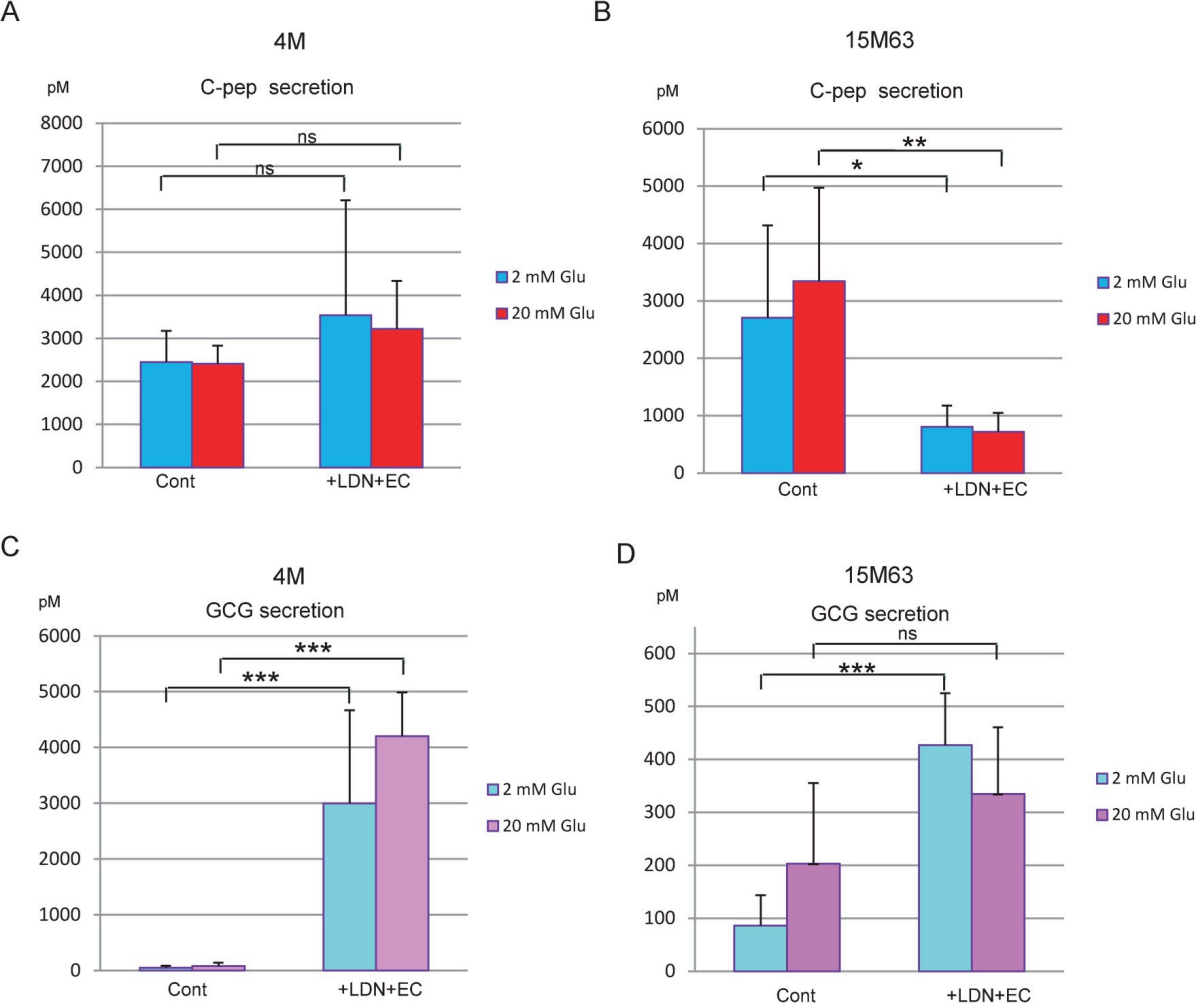
Hormone secretion experiments (A, B) Analysis of insulin secretion from cells stimulated by low or high glucose. A; 4M, B; 15M63 (C, D) Analysis of glucagon secretion from the cells stimulated by low or high glucose. C; 4M, D; 15M63, Experiments were performed three times. *P<0.05, **P<0.01, ***P<0.001. ns: not significant.

Hormone secretion in response to glucose stimulation is a very important function of pancreatic endocrine cells. We performed hormone secretion experiments by stimulating the differentiated cells with different concentrations of glucose. Usually, high glucose stimulates insulin secretion but represses glucagon secretion; in contrast, low glucose enhances glucagon secretion but not insulin secretion. For 4M, the amount of insulin secretion was not significantly different between control and LDN + EC cells [Fig 4A], but a remarkable difference was seen in GCG secretion, which was markedly enhanced in LDN + EC cells compared with control cells [Fig 4C]. In 15M63 cells, the amount of insulin secretion was extremely low in LDN + EC cells compared with control cells [Fig 4B]; however, there wasn't much difference in GCG secretion between control and LDN + EC cells in 20mM glucose [Fig 4D]. These results are consistent with the aforementioned data obtained by RT-PCR and immunocytochemistry. Although hormone secretion was detected, it was very high in terms of glucose responsiveness.

### Transplantation of LDN + EC cells in vivo

**Fig 5:**
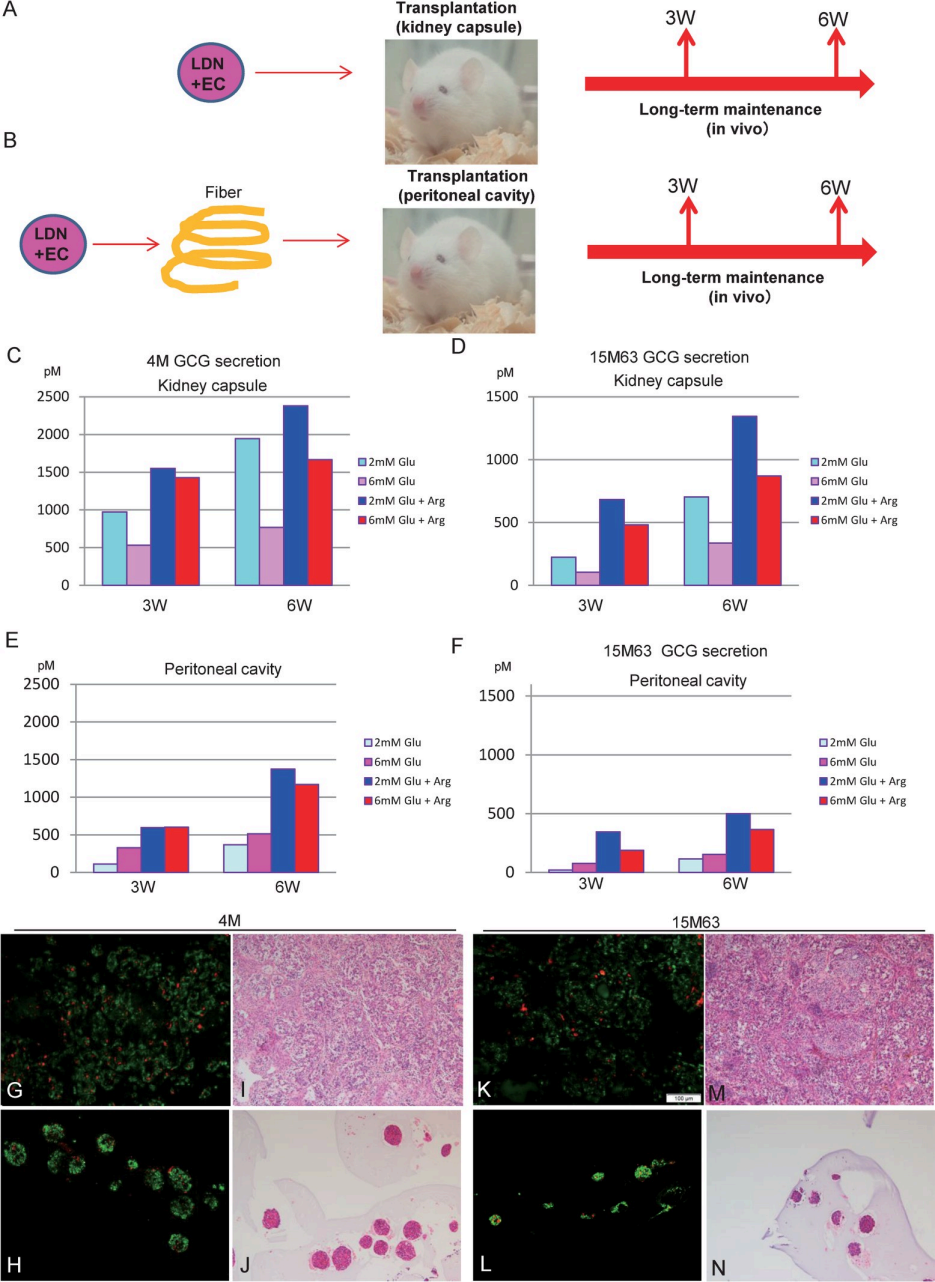
Scheme of long-term maintenance of LDN + EC cells and GCG secretion assay (A) LDN+ EC cells were transplanted under the kidney capsule and retrieved at 3 or 6 weeks after transplantation. (B) LDN + EC cells embedded in alginate fiber were implanted into the peritoneal cavity and retrieved at the same timepoint. (C-F) Analysis of glucagon secretion from the cells stimulated with 1- and 6-mM glucose with/without arginine. (C, E) cells transplanted under the kidney capsule. D; 4M, E; 15M63 (D, F) cells embedded in alginate fiber implanted into the peritoneal cavity. G; 4M, E; 15M63 (G, K) Immunochemistry for LDN + EC cells transplanted under the kidney capsule. G; 4M (4 weeks), K; 15M63; (6weeks). (H, L) Immunochemistry for LDN + EC cells embedded alginate fiber implanted into the peritoneal cavity. H; 4M (4 weeks), L; 15M63 (4 weeks). Red; insulin c-peptide, green; glucagon. The rates of glucagon positive cells (88.3 ± 2.0%) and C-peptide positive cells (5.4 ± 0.04%) in alginate fiber for 4M. The rates of glucagon positive cells (91.2 ± 3.4%) and C-peptide positive cells (3.2 ± 0.5%) in alginate fiber for 15M63. (I, M) HE staining for LDN + EC cells transplanted under the kidney capsule. I; 4M (4 weeks), M; 15M63; (6weeks). (J, N) HE staining for LDN + EC cells embedded alginate fiber implanted into the peritoneal cavity. J; 4M (4 weeks), N; 15M63 (4 weeks). The rate of survived cells was 94.8 ± 2.6% in alginate fiber for 4M and 83.2 ± 11.4% for 15M63 respectively. N = 2 each.

A previous study reported that human pancreatic beta cells differentiated from ESCs/iPSCs further matured in vivo after transplantation into rodents [20]. Therefore, we tested whether LDN + EC cells achieved glucose responsive glucagon secretion *in vivo*. We compared the secretory function of cells under two different conditions. One was a direct transplantation under the kidney capsule [Fig 5A]. The other was an indirect transplantation into the peritoneal cavity after encapsulating cells in alginate [Fig 5B]. We embedded the cells into alginate gel and formed a fiber, as previously described [24]. The encapsulated cells did not disperse in the peritoneal cavity, and they survived stably for a long time *in vivo*. Three or six weeks after implantation, we performed nephrectomies, and the transplanted cells were resected en bloc. Alginate fiber was retrieved from the peritoneal cavity at the same timepoint. Then we evaluated glucagon secretion *ex vivo* using ELISA. We adopted ex vivo analysis to avoid any mouse-derived GCG secretion, because the current ELISA does not distinguish between human GCG and mouse GCG. As blood glucose increases, GCG secretion from pancreatic alpha cells is usually inhibited *in vivo* up to 6mM. However, a higher concentration of glucose (>15mM) is reported to stimulate GCG secretion from intact islet or isolated alpha cells *in vitro* [25–29]. Considering these studies, we performed GCG secretion experiments with 1 mM or 6 mM glucose. Cells transplanted under the kidney capsule secreted larger amounts of GCG than alginate encapsulated cells [Fig 5C–5E], and cells transplanted for 6 weeks secreted more GCG than those for 3 weeks [Fig 5C and 5D]. Moreover, GCG secretion was more suppressed in 6mM glucose comparing with levels in 1mM glucose. Addition of arginine stimulated GCG secretion both in 1mM and 6mM glucose [Fig 5C and 5D]. These results suggest that these cells displayed normal glucose responsive GCG secretion for alpha cells. Encapsulated cells from the peritoneal cavity secreted lower amounts of GCG and didn’t suppress GCG secretion in 6mM glucose, although GCG secretion was up-regulated by arginine stimulation [Fig 5E and 5F]. These data indicate that cells directly transplanted under the kidney capsule were superior to cells encapsulated in alginate fiber in peritoneal cavity. Immunochemistry for transplanted cells confirmed that almost all the cells differentiated into pancreatic alpha cells in both the kidney [Fig 5G and 5K] and the peritoneal cavity. The rate of glucagon positive cells and C-peptide positive cells in alginate fiber for 4M was 88.3 ± 2.0%, 5.4 ± 0.04%, respectively. The rate of glucagon positive cells and C-peptide positive cells in alginate fiber for 15M63 was 91.2 ± 3.4%, 3.2 ± 0.5%, respectively. [Fig 5H–5L]. For cells transplanted under the kidney capsule, it was difficult to calculate positive cells rate exactly, because the extirpated grafts contained mouse cells. However, the glucagon positive cells are a gross majority compared with C-peptide positive cells in cells transplanted under the kidney capsules as well as in alginate fibers. HE staining revealed that no dead cells were observed on the renal sample (Fig 5I–5M), and the rates of surviving cells were 94.8 ± 2.6% in alginate fiber for 4M and 83.2 ± 11.4% for 15M63 respectively (Fig 5J–5N).

## Discussion

In the present study, we demonstrated that the differentiation of pancreatic alpha cells from hPSCs was determined by the addition of LDN193189 and EC23 during stage 2 (between definitive endoderm and primitive gut tube). Using two different hPSCs lines, our protocol allowed us to reduce NKX6.1 expression and differentiated almost all the cells into pancreatic alpha cells *in vivo*. Our study demonstrates that NKX6.1 expression can be controlled by the timing of BMP antagonism and activation of retinoic acid signaling during the pancreatic differentiation process.

To date, only two papers have reported methods of generation of pancreatic alpha cells from hPSCs [21,22]. Rezania et al. first reported functional GCG secreting cells produced from human ES cells. They used cyclopamine (sonic hedgehog inhibitor) during stage 2 and added noggin (BMP inhibitor) during stage 3; the cells differentiated into pancreatic alpha cells *in vivo* after transplantation [21]. They also noted that removal of noggin (BMP antagonist) from stage 5 (Immature Endocrine) might confer sufficient BMP signaling to promote the formation and maturation of pancreatic alpha cells. Although their methods for generating low NKX6.1 expressing cells were not described in detail, NKX6.1 negative or low cells did differentiate into pancreatic alpha cells *in vivo* after transplantation [15], which is consistent with our results.

Melton`s lab has very recently reported that downregulating NKX6.1 expression by using BMP signaling antagonist LDN after the gut tube endoderm stage (step3) led hES cells to become pancreatic alpha cells [22]. They reported that removal of KGF and SANT1 and addition of LDN193189 at day 2 of stage 3 reduced NKX6.1 expression and that this protocol produced over 60% of insulin+ and glucagon+ coexpressing pre-alpha cells. They also reported that these pre-alpha cells continued to express insulin and glucagon proteins for 2 weeks after transplantation, but, at six weeks, they persisted in expressing glucagon but not insulin. Although their timing of adding LDN193189 and retinoic acid differed from our protocol, we can at least conclude that inhibition of BMP signaling plays an important role in NKX6.1 expression during pancreatic endocrine cell differentiation. In fact, it has been reported that BMP signaling is needed for generation of NKX6.1 positive pancreatic progenitor [30].

Many differentiation protocols for pancreatic beta cells have included inhibition of BMP signaling [11–20,31,32], because it suppresses hepatic lineage differentiation and promotes pancreatic endocrine differentiation [17,33]. Even when our and Melton`s previous differentiation protocols contained BMP antagonism, functional pancreatic islet-like cells were induced from hPSCs [16,20]. In our protocol, BMP antagonism by LDN or dorsomorphin during stage 2 markedly reduced NKX6.1 expression, but addition of BMP4 exogenously did not enhance NKX6.1 expression, indicating that differentiating cells secrete sufficient BMP ligand, that BMP signaling at stage 2 is essential for high NKX6.1 expression, and that regulation of NKX6.1 expression by BMP signaling depends on the time window.

Although LDN + EC cells did not display glucose responsive GCG secretion in *in vitro* differentiation, they correctly responded to glucose stimulation after transplantation under the kidney capsule, as shown in Fig 5C and 5D. Several studies have also reported that alpha cells are formed or enriched by transplanting them under the kidney capsule [21,22,34,35]. We observed a big difference in GCG secretion between directly transplanted cells and encapsulated cells in terms of glucose responsiveness *in vivo*. Cells transplanted under the kidney capsule responded correctly to glucose stimulation, but cells encapsulated in alginate fiber did not. One of the reasons for this difference might be vascularization, because islets are highly vascularized *in vivo* [36]; vascularization effectively occurred in transplanted cells in the kidney but not in the peritoneal cavity. Vascularization has been shown to be an important factor for islet function after transplantation [37]. Further study will be needed to elucidate the effects of oxygen tension and interaction with endothelial cells on islet cells.

Although several humoral maturation promoting factors for islet cells have been suggested, including thyroid hormone, sex steroid and Apolipoprotein E [38–41], the difference between transplanted cells in the kidney and encapsulated cells in the peritoneal cavity cannot be explained by such factors, because soluble factors can reach encapsulated cells in the peritoneal cavity. Extracellular matrix is also supposed to be an important factor for maturation and function of islet cells [42–44]. Cells directly implanted in the kidney were allowed contact with ECM as well as with endothelial cells; encapsulated cells in alginate fiber were not. Strictly speaking, we have to consider the transplantation site for comparison. It would have been desirable to implant the cells into the peritoneal cavity without alginate fiber as a control, but it is difficult to retrieve the implanted cells because they disperse after transplantation. Culture time is also an important factor for maturation of cells; the amount of GCG secretion increased from 3 to 6 W *in vivo*, as shown in Fig 5C and 5D. It may be possible to maturate alpha cells *in vitro* by improving culture conditions, because Peterson et al. reported that PKC activator PDBu promoted the differentiation into alpha cells in vitro [22]. Therefore, PKC signaling might be activated in transplanted cells in the kidney.

**Fig 6:**
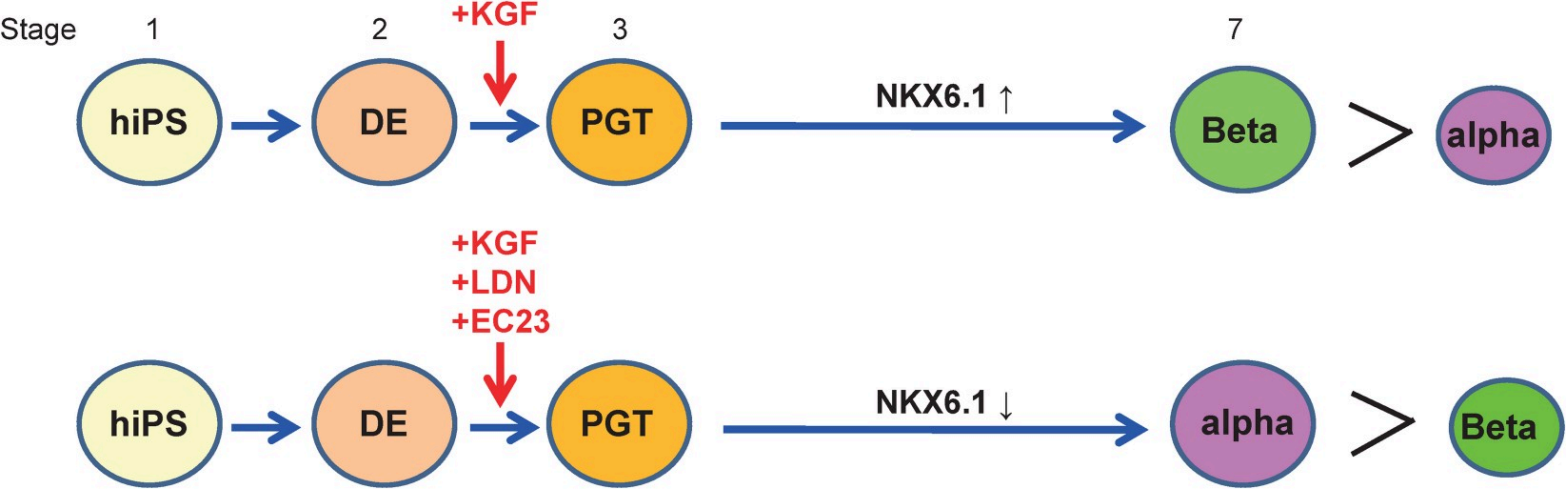
Scheme of differentiation of LDN + EC cells. Treatment with LDN193189 and EC23 at stage 3 suppressed NKX6.1 expression. Ultimately, these cells differentiated into pancreatic alpha cells.

In this research, we used two hiPSCs lines, 4M and 15M63, and observed differences in gene expression patterns for insulin and glucagon during the differentiation process. GCG expression was significantly different between control and LDN + EC cells in 4M [Fig 3E], but not in 15M63 d[Fig 3F]. In contrast, insulin expression was significantly different between control and LDN + EC cells in 15M63 [Fig 3D], but not in 4M [Fig 3C]. These data suggest that the treatment of LDN + EC clearly enhanced alpha cell differentiation for 4M and inhibited beta cell differentiation for 15M63 during in vitro differentiation. Although variable differentiation propensities among hPSCs lines has been reported [45], NKX6.1 expression was remarkably reduced in LEN + EC cells in both hiPSCs lines. Importantly, even if the effect of the treatment of LDN + EC on related gene expression varied between iPSCs lines in vitro, almost all the cells finally differentiated into pancreatic alpha cells after transplantation in vivo. These results indicate that LDN + EC cells have the potential to differentiate into pancreatic alpha cells in in vivo situations [Fig 6].

In conclusion, it is important to suppress NKX6.1 expression for efficient induction of pancreatic alpha cells, using the combination of LDN193189 and EC23 with proper timing. After developing methods for isolating human pancreatic alpha cells, this discovery will be useful for study of behaviors such as the mechanism of glucagon secretion and the pathophysiology of hyperglucagonemia in diabetes patients.

## Supporting information

S1 Checklist The ARRIVE guidelines 2.0: Author checklist. (PDF)

S1 Fig Expression of NKX6.1 in cells treated by inhibition or activation of BMP signaling by qPCR. (A, B) NKX6.1 expression in cells treated with BMP antagonist dorsomorphin (DM) or LDN193189. A; 4M, B; 15M63 (C, D) NKX6.1 expression in cells treated with BMP4. C; 4M, D; 15M63. N = 2 each **P<0.01, ***P<0.001. ns: not significant.
(EPS)

S2 Fig Morphology of spheroids of control or LDN + EC cells. A: LDN treated cells in 4M. B: LDN + EC treated cells in 4M. C: LDN treated cells in 15M63. D: LDN + EC treated cells in 15M63.
(EPS)
